# Endoscopic Double Stenting for the Management of Combined Malignant Biliary and Duodenal Obstruction

**DOI:** 10.3390/jcm10153372

**Published:** 2021-07-29

**Authors:** Tsuyoshi Takeda, Takashi Sasaki, Takeshi Okamoto, Naoki Sasahira

**Affiliations:** Department of Hepato-Biliary-Pancreatic Medicine, Cancer Institute Hospital of Japanese Foundation for Cancer Research, 3-8-31, Ariake, Koto-ku, Tokyo 135-8550, Japan; tsuyoshi.takeda@jfcr.or.jp (T.T.); takeshi.okamoto@jfcr.or.jp (T.O.); naoki.sasahira@jfcr.or.jp (N.S.)

**Keywords:** biliary obstruction, duodenal obstruction, double stenting, anti-reflux metal stent, lumen-opposing metal stent

## Abstract

Periampullary cancers are often diagnosed at advanced stages and can cause both biliary and duodenal obstruction. As these two obstructions reduce patients’ performance status and quality of life, appropriate management of the disease is important. Combined malignant biliary and duodenal obstruction is classified according to the location and timing of the duodenal obstruction, which also affect treatment options. Traditionally, surgical bypass (gastrojejunostomy and hepaticojejunostomy) has been performed for the treatment of unresectable periampullary cancer. However, it has recently been substituted by less invasive endoscopic procedures due to its high morbidity and mortality. Thus, endoscopic double stenting (transpapillary stenting and enteral stenting) has become the current standard of care. Limitations of transpapillary stenting include its technical difficulty and the risk of duodenal-biliary reflux. Recently, endoscopic ultrasound-guided procedures have emerged as a novel platform and have been increasingly utilized in the management of biliary and duodenal obstruction. As the prognosis of periampullary cancer has improved due to recent advances in chemotherapy, treatment strategies for biliary and duodenal obstruction are becoming more important. In this article, we review the treatment strategies for combined malignant biliary and duodenal obstruction based on the latest evidence.

## 1. Introduction

Periampullary cancers, including pancreatic cancer, biliary tract cancer, duodenal cancer and ampullary cancer, are often diagnosed at advanced stages and can cause both biliary and duodenal obstruction. Biliary obstruction may lead to cholangitis or liver dysfunction, whereas duodenal obstruction may present with decreased oral intake, nausea and vomiting. These two obstructions reduce patients’ performance status and quality of life and may deprive them of the opportunity to receive antitumor treatment. Therefore, appropriate treatment and management are very important.

Traditionally, double surgical bypass (gastrojejunostomy and hepaticojejunostomy) has been performed for the treatment of combined biliary and duodenal obstruction in patients with unresectable periampullary cancer [1,2,3]. Endoscopic double stenting (transpapillary stenting and enteral stenting) has become the standard treatment due to its lower invasiveness and shorter recovery time [4]. Percutaneous transhepatic biliary drainage (PTBD) has been widely used as an alternative treatment after failed endoscopic retrograde cholangiopancreatography (ERCP), but it has disadvantages such as skin infection, pain and decreased quality of life. Recently, endoscopic ultrasound (EUS)-guided procedures have emerged as a novel platform and have been increasingly utilized in the management of biliary and duodenal obstruction. As the prognosis of periampullary cancer has improved due to recent advances in chemotherapy, treatment strategies for biliary and duodenal obstruction are becoming more important. In this article, we review the treatment strategies for combined malignant biliary and duodenal obstruction based on the latest evidence.

## 2. Classification of Combined Malignant Biliary and Duodenal Obstruction

Combined malignant biliary and duodenal obstruction has been classified according to the location and timing of the duodenal obstruction (Table 1) [5]. First, duodenal obstruction can be categorized into three types based on the location relative to the major papilla: type I, duodenal obstruction proximal to the major papilla; type II, duodenal obstruction involving the major papilla; and type III, duodenal obstruction distal to the major papilla. Double stenting is most technically challenging in patients with type II obstruction because transpapillary biliary access is difficult, if not impossible [5]. Transpapillary biliary stenting may not be difficult in patients with type I obstruction if the scope can pass through the duodenal stricture after dilation of the duodenal stricture or placement of a duodenal stent [6,7]. Transpapillary biliary stenting in patients with type III obstruction may also be easy to manage because the major papilla is located proximal to the duodenal stricture. However, these types face a risk of duodenal-biliary reflux [8]. Such patients are good candidates for EUS-guided biliary drainage (EUS-BD) [9,10,11,12].

Second, biliary obstruction can be classified into three groups according to the timing of duodenal and biliary obstruction: group 1, biliary obstruction occurring before the onset of duodenal obstruction; group 2, biliary obstruction occurring simultaneously with duodenal obstruction; and group 3, biliary obstruction occurring after the onset of duodenal obstruction. Group 1 is the most common, followed by group 3 and group 2. In group 1, the type of previously inserted biliary stent could affect the treatment strategy. The introduction of covered biliary self-expandable metallic stents (SEMS) has broadened the range of treatment options available due to its removability. Both classifications are important in determining the optimal management strategy for combined biliary and duodenal obstructions.

Combined biliary and duodenal obstruction also occurs in patients with surgically altered anatomy. However, evidence is scarce in this area. One study proposed a new classification for malignant afferent loop obstruction according to the location of the intestinal stricture in relation to the major papilla or bilioenteric anastomosis [13]: type 1, obstruction site located distal to the major papilla or bilioenteric anastomosis; type 2, obstruction site involving the major papilla or bilioenteric anastomosis; and type 3, obstruction site located between bilioenteric and pancreaticoenteric anastomoses. Recently, enteral stenting employing the through-the-scope technique with a short-type balloon-assisted enteroscope and SEMS with a 9-Fr delivery system has become possible [13,14,15,16]. Nevertheless, endoscopic biliary stenting remains technically demanding due to difficulties in achieving biliary access. A combination of PTBD or EUS-BD may be required in these situations.

## 3. Treatment Options for Combined Malignant Biliary and Duodenal Obstruction

### 3.1. Surgical Approach

Traditionally, double surgical bypass (gastrojejunostomy and hepaticojejunostomy) has been performed for symptomatic treatment of unresectable periampullary cancer [1,2,3]. However, it has recently been substituted by less invasive endoscopic procedures due to its high morbidity and mortality. A recent systematic review and meta-analysis reported that endoscopic double stenting was associated with higher clinical success (97% vs. 86%) and less adverse events (13% vs. 28%), but with a more frequent need for reintervention (21% vs. 10%) compared with double surgical bypass [17]. Even though endoscopic double stenting has become the standard treatment for combined biliary and duodenal obstruction [18], minimally invasive surgical procedures such as laparoscopic gastrojejunostomy are still favored in patients with a long life expectancy, due to reports suggesting better long-term outcomes [19,20,21]. On the other hand, data on the efficacy of endoscopic duodenal stenting for patients with long life expectancy are also increasing [22,23,24]. In addition, EUS-guided gastroenterostomy (EUS-GE) has recently been developed as a novel technique for the management of gastric outlet obstruction, with promising results [25,26,27,28]. Further research is needed to determine the optimal management for this population.

### 3.2. Percutaneous Approach

PTBD including percutaneous transhepatic biliary stenting is a well-established rescue procedure for the palliation of malignant biliary obstruction [29], especially when the endoscopic transpapillary approach is not possible. However, this procedure carries high morbidity. EUS-BD is currently gaining wide acceptance among experienced endosonographers. A multicenter randomized trial reported that procedure-related adverse events were significantly higher in PTBD than in EUS-BD (31.2% vs. 8.8%), with similar efficacy [30]. EUS-BD may be preferrable when transpapillary biliary stenting is unsuccessful, if expertise is available.

### 3.3. Endoscopic Approach

Endoscopic double stenting is the current standard treatment for combined biliary and duodenal obstruction. For malignant biliary obstruction, transpapillary biliary drainage via ERCP and EUS-BD are the two major treatment options. Studies reporting outcomes of endoscopic double stenting including at least 10 subjects are summarized in Table 2. We reclassified biliary drainage procedures that required percutaneous techniques, including PTBD rendezvous technique and percutaneous transhepatic SEMS insertion, as technical failures with respect to endoscopic biliary drainage. In general, the technical success rate was greatly influenced by the biliary drainage method and the proportion of type II obstructions. A systematic review and meta-analysis found that ERCP was associated with similar clinical success and less adverse events (3% vs. 23%) compared to EUS-BD for biliary drainage as part of double stenting [17]. As a result, ERCP remains the preferred treatment option when transpapillary biliary access is possible. While EUS-BD is generally considered a salvage technique for difficult or failed ERCP [31,32], two recent randomized controlled trials reported similar adverse event rates (21.2% vs. 14.7%) in expert hands [33,34].

EUS-BD is especially useful in patients with type II obstruction because transpapillary biliary access is difficult. A retrospective study reported that the technical success rate of EUS-BD was significantly higher than that of transpapillary biliary drainage (95.2% vs. 56.0%) in pancreatic cancer patients with an indwelling duodenal stent [35]. Furthermore, duodenal obstruction has been reported as a risk factor for early transpapillary biliary SEMS dysfunction due to duodenal-biliary reflux [36,37]. Therefore, these two situations are good indications for EUS-BD. The two major EUS-BD techniques are EUS-guided hepatico-gastrostomy (EUS-HGS) and choledocho-duodenostromy (EUS-CDS). A retrospective study comparing the efficacy and safety of EUS-HGS with EUS-CDS suggested that EUS-HGS may be superior to EUS-CDS, with longer stent patency (biliary stent patency: median 133 days vs. 37 days) and fewer adverse events [38]. EUS-CDS was particularly associated with reflux cholangitis, probably due to the closer distance between the duodenal stent and the bilioduodenal fistula relative to EUS-HGS. A recent multicenter randomized controlled study comparing the efficacy and safety of EUS-HGS with EUS-CDS demonstrated that the clinical success, stent patency and adverse events were similar between the two procedures [39]. In summary, disadvantages of EUS-CDS include susceptibility to duodenal-biliary reflux and difficult access in type I obstruction, while those of EUS-HGS include the inability to puncture a non-dilated left intrahepatic bile duct and SEMS occlusion due to bile duct hyperplasia.

**Table 2:** Results of endoscopic double stenting for combined malignant biliary and duodenal obstruction.

Study	*N*	Biliary Drainage	Biliary Stent Type	Technical Success (%)		Early Adverse Events
				Biliary Stent	Duodenal Stent	
Kaw et al. [40]	18	ERCP	SEMS	94	94	Bleeding 1
Vanbiervliet et al. [41]	18	ERCP	SEMS	94	Indwelling	None
Maire et al. [42]	23	ERCP	PS, SEMS	91	96	None
Mutignani et al. [5]	64	ERCP	PS, SEMS	97	100	Pancreatitis 1, cholangitis 1, cholecystitis 1, bleeding 1
Kim et al. [4]	24	ERCP	PS, SEMS	54	100	Pancreatitis 3, cholangitis 1
Tonozuka et al. [11]	11	ERCP, EUS-BD	SEMS	100	100	None
Khashab et al. [43]	38	ERCP, EUS-BD	PS, SEMS	66	Indwelling	Cholangitis 1
Yu et al. [44]	17	ERCP	SEMS	100	100	Bleeding 1
Canene et al. [45]	50	ERCP	SEMS	84	100	NA
Hamada et al. [36]	20	ERCP, EUS-BD	PS, SEMS	100	Indwelling	Bleeding 1, pancreatitis 1
Manta et al. [46]	15	ERCP, EUS-BD	SEMS	87	100	None
Ogura et al. [38]	39	EUS-BD	SEMS	100	100	None
Sato et al. [9]	50	ERCP, EUS-BD	SEMS	86	100	NA
Matsumoto et al. [10]	81	ERCP, EUS-BD	PS, SEMS	100	100	NA
Hamada et al. [12]	110	ERCP, EUS-BD	PS, SEMS	100	100	NA
Hori et al. [47]	109	ERCP	SEMS	93	99	Pneumonia 2, pancreatitis 1
Staub et al. [6]	71	ERCP	PS, SEMS	85	Indwelling	Cholangitis 2, perforation 1
Yamao et al. [35]	39	ERCP, EUS-BD	PS, SEMS	87	Indwelling	NA
Debourdeau et al. [48]	31	ERCP, EUS-BD	SEMS	65	100	NA
Mangiavillano et al. [49]	23	EUS-BD, EUS-GBD	SEMS	96	100	None

*N*, number; ERCP, endoscopic retrograde cholangiopancreatography; EUS-BD, endoscopic ultrasound-guided biliary drainage; EUS-GBD, endoscopic ultrasound-guided gallbladder drainage; PS, plastic stent; SEMS, self-expandable metallic stent; NA, not available.

### 3.4. Novel Types of Stents

#### 3.4.1. Anti-Reflux Metal Stents

Several types of anti-reflux metal stents (ARMS) have been made to prevent duodenal-biliary reflux [50,51,52,53,54,55,56]. Although ARMS was associated with a lower rate of stent occlusion compared to conventional SEMS in several studies on distal malignant biliary obstruction, the results were inconsistent and stent patency rates were low. Recently, two retrospective studies showed that a novel duckbill-type ARMS was more effective in preventing duodenal-biliary reflux than conventional SEMS [57,58]. ARMS may be effective not only for transpapillary biliary stenting, but also for EUS-CDS in patients with combined biliary and duodenal obstruction [59]. Prospective studies are needed to further evaluate the efficacy and safety of AMRS especially in the setting of combined biliary and duodenal obstruction.

#### 3.4.2. Lumen-Apposing Metal Stents

Lumen-apposing metal stents (LAMS), designed for transluminal drainage of nonadherent lumens, were first reported by Binmoeller and Shah in 2011 [60]. Although this stent was initially created for drainage of pancreatic fluid collections, use of LAMS has been reported in gallbladder drainage, biliary drainage (EUS-CDS) and the creation of gastrointestinal fistulae [61]. Recently, a retrospective study reported the technical feasibility of LAMS insertion through the mesh of an indwelling duodenal stent with a technical success rate of 95.6% in 23 patients [49]. Prospective studies with larger sample sizes are needed to further evaluate these LAMS applications.

### 3.5. EUS-GE

EUS-GE using LAMS has recently received attention as a new alternative for the treatment of gastric outlet obstruction. Several techniques including the direct technique, the device-assisted technique and EUS-guided double balloon-occluded gastrojejunostomy bypass have been reported [62,63,64,65,66]. Each technique involves the LAMS being placed between the stomach and the small intestine distal to the obstructed bowel under EUS and fluoroscopic guidance. Limitations of the traditional approaches (surgical bypass and enteral stent placement) include surgical morbidity and risk of stent occlusion due to tumor ingrowth/overgrowth. Potential advantages of EUS-GE over traditional approaches include less invasiveness (versus surgery) and longer stent patency (versus enteral stent placement). An international, multicenter, retrospective study comparing EUS-GE with laparoscopic GE showed that EUS-GE had similar technical and clinical success rates with reduced time to oral intake, shorter hospital duration and fewer adverse events [67]. A systematic review and meta-analysis comparing EUS-GE and enteral stenting showed that EUS-GE was associated with a significantly lower rate of reintervention despite a comparable technical/clinical success and safety profile [68]. A systematic review and meta-analysis comparing EUS-GE with surgical bypass and enteral stenting demonstrated that EUS-GE was associated with improved outcomes compared to enteral stenting and with shorter hospital stays compared to surgical bypass.

Several case reports have also described the efficacy of EUS-GE in combination with EUS-BD for the management of combined biliary and duodenal obstruction [69,70,71]. Important advantages of these EUS-guided procedures are the ability to bypass the tumor, reducing the risk of stent occlusion due to tumor ingrowth/overgrowth. Thus, a combination of EUS-BD and EUS-GE may become the optimal procedure for combined biliary and duodenal obstruction in the future. However, several issues remain unresolved. First, EUS-GE is technically challenging, requiring considerable expertise in both EUS and ERCP. Second, development of dedicated accessories and standardization of the procedure are needed for widespread use. Third, EUS-GE may be technically difficult when malignancies invade the fourth part of the duodenum or the jejunum near the ligament of Treitz. Fourth, EUS-GE is contraindicated in patients with significant ascites.

## 4. Treatment Strategies for Combined Malignant Biliary and Duodenal Obstruction

Based on the above-mentioned evidence, transpapillary stenting and enteral stenting is currently the standard option, whereas to date, EUS-guided procedures are generally reserved for failed or refractory cases to conventional stenting. EUS-GE is especially reserved for selected specialized high-volume centers with extensive experience.

In type I obstruction, transpapillary stenting is possible if the endoscope can pass through the duodenal stricture or an indwelling duodenal stent. Dilation of the duodenal stricture by a balloon or insertion of a duodenal stent prior to ERCP can facilitate scope insertion. When transpapillary stenting fails, EUS-HGS is the next preferred option. Adding EUS-antegrade stenting to EUS-HGS may allow for longer stent patency [72,73].

In type II obstruction, transpapillary stenting is very difficult because the duodenal obstruction involves the major papilla. Although there are several techniques for transpapillary biliary access including RV techniques under PTBD or EUS guidance, success rates are suboptimal. Furthermore, type II obstruction is reported to be susceptible to duodenal-biliary reflux. Double stenting with EUS-HGS or EUS-CDS using ARMS are potential solutions to overcome this issue.

In type III obstruction, transpapillary stenting is not hindered by duodenal obstruction, which is located distal to the major papilla. As with type II obstruction, type III obstruction is reported to present a high risk of duodenal-biliary reflux. Transpapillary stenting using ARMS may be preferable in this context. EUS-HGS or EUS-CDS using ARMS are also possible alternatives in this scenario.

## 5. Conclusions

Endoscopic double stenting (transpapillary stenting and enteral stenting) is the current standard of care for combined biliary and duodenal obstruction. However, reports on the usefulness of EUS-guided procedures have recently been increasing. An important advantage of EUS-guided procedures is the ability to create a fistula away from the obstructing tumor. With the development of dedicated devices and standardization of the procedure, EUS-guided procedures including EUS-HGS, EUS-CDS and EUS-GE can potentially become the standard of care treatment in the future. The development of new stent types, including ARMS and LAMS, also plays an important role in the management of combined biliary and duodenal obstruction.

## Figures and Tables

**Table 1:** Classification of combined malignant biliary and duodenal obstruction.

Location	
Type I	Duodenal obstruction proximal to the major papilla
Type II	Duodenal obstruction involving the major papilla
Type III	Duodenal obstruction distal to the major papilla
Timing	
Group 1	Biliary obstruction occurring before the onset of duodenal obstruction
Group 2	Biliary and duodenal obstruction occurring simultaneously
Group 3	Biliary obstruction occurring after the onset of duodenal obstruction

## Data Availability

Not applicable.

## References

[1] Bartlett E.K., Wachtel H., Fraker D.L., Vollmer C.M., Drebin J.A., Kelz R.R., Karakousis G.C., Roses R.E. (2014). Surgical palliation for pancreatic malignancy: Practice patterns and predictors of morbidity and mortality. J. Gastrointest. Surg..

[2] Kohan G., Ocampo C.G., Zandalazini H.I., Klappenbach R., Yazyi F., Ditulio O., Coturel A., Canullán C., Porras L.T.C., Rodriguez J.A. (2015). Laparoscopic hepaticojejunostomy and gastrojejunostomy for palliative treatment of pancreatic head cancer in 48 patients. Surg. Endosc..

[3] Lyons J.M., Karkar A., Correa-Gallego C.C., D’Angelica M.I., DeMatteo R.P., Fong Y., Kingham T.P., Jarnagin W.R., Brennan M.F., Allen P.J. (2012). Operative procedures for unresectable pancreatic cancer: Does operative bypass decrease requirements for postoperative procedures and in-hospital days?. HPB.

[4] Kim K.O., Kim T.N., Lee H.C. (2012). Effectiveness of combined biliary and duodenal stenting in patients with malignant biliary and duodenal obstruction. Scand. J. Gastroenterol..

[5] Mutignani M., Tringali A., Shah S.G., Perri V., Familiari P., Iacopini F., Spada C., Costamagna G. (2007). Combined endoscopic stent insertion in malignant biliary and duodenal obstruction. Endoscopy.

[6] Staub J., Siddiqui A., Taylor L.J., Loren D., Kowalski T., Adler D.G. (2018). ERCP performed through previously placed duodenal stents: A multicenter retrospective study of outcomes and adverse events. Gastrointest. Endosc..

[7] Yao J.F., Zhang L., Wu H. (2016). Analysis of high risk factors for endoscopic retrograde cholangiopancreatography biliary metallic stenting after malignant duodenal stricture SEMS implantation. J. Biol. Regul. Homeost. Agents.

[8] Hamada T., Nakai Y., Isayama H., Sasaki T., Kogure H., Kawakubo K., Sasahira N., Yamamoto N., Togawa O., Mizuno S. (2013). Duodenal metal stent placement is a risk factor for biliary metal stent dysfunction: An analysis using a time-dependent covariate. Surg. Endosc..

[9] Sato T., Hara K., Mizuno N., Hijioka S., Imaoka H., Yogi T., Tsutsumi H., Fujiyoshi T., Niwa Y., Tajika M. (2016). Type of Combined Endoscopic Biliary and Gastroduodenal Stenting Is Significant for Biliary Route Maintenance. Intern. Med..

[10] Matsumoto K., Kato H., Tsutsumi K., Mizukawa S., Yabe S., Seki H., Akimoto Y., Uchida D., Tomoda T., Yamamoto N. (2017). Long-term outcomes and risk factors of biliary stent dysfunction after endoscopic double stenting for malignant biliary and duodenal obstructions. Dig. Endosc..

[11] Tonozuka R., Itoi T., Sofuni A., Itokawa F., Moriyasu F. (2013). Endoscopic double stenting for the treatment of malignant biliary and duodenal obstruction due to pancreatic cancer. Dig. Endosc..

[12] Hamada T., Nakai Y., Lau J.Y., Moon J.H., Hayashi T., Yasuda I., Hu B., Seo D.W., Kawakami H., Kuwatani M. (2018). International study of endoscopic management of distal malignant biliary obstruction combined with duodenal obstruction. Scand. J. Gastroenterol..

[13] Sasaki T., Yamada I., Matsuyama M., Sasahira N. (2018). Enteral stent placement for malignant afferent loop obstruction by the through-the-scope technique using a short-type single-balloon enteroscope. Endosc. Int. Open.

[14] Shimatani M., Takaoka M., Tokuhara M., Kato K., Miyoshi H., Ikeura T., Okazaki K. (2016). Through-the-scope self-expanding metal stent placement using newly developed short double-balloon endoscope for the effective management of malignant afferent-loop obstruction. Endoscopy.

[15] Minaga K., Kitano M., Takenaka M. (2016). Through-the-scope enteral metal stent placement using a short-type single-balloon enteroscope for malignant surgically reconstructed jejunal stenosis (with video). Dig. Endosc..

[16] Tsutsumi K., Kato H., Okada H. (2017). Impact of a Newly Developed Short Double-Balloon Enteroscope on Stent Placement in Patients with Surgically Altered Anatomies. Gut Liver.

[17] Fábián A., Bor R., Gede N., Bacsur P., Pécsi D., Hegyi P., Tóth B., Szakács Z., Vincze Á., Ruzsics I. (2020). Double Stenting for Malignant Biliary and Duodenal Obstruction: A Systematic Review and Meta-Analysis. Clin. Transl. Gastroenterol..

[18] Moon J.H., Choi H.J. (2011). Endoscopic double-metallic stenting for malignant biliary and duodenal obstructions. J. Hepatobiliary Pancreat Sci..

[19] Jeurnink S.M., Steyerberg E.W., van Hooft J.E., van Eijck C.H., Schwartz M.P., Vleggaar F.P., Kuipers E.J., Siersema P.D., Dutch Sustent Study Group (2010). Surgical gastrojejunostomy or endoscopic stent placement for the palliation of malignant gastric outlet obstruction (SUSTENT study): A multicenter randomized trial. Gastrointest. Endosc..

[20] Manuel-Vázquez A., Latorre-Fragua R., Ramiro-Pérez C., López-Marcano A., la Plaza-Llamas R., Ramia J.M. (2018). Laparoscopic gastrojejunostomy for gastric outlet obstruction in patients with unresectable hepatopancreatobiliary cancers: A personal series and systematic review of the literature. World J. Gastroenterol..

[21] Min S.H., Son S.Y., Jung D.H., Lee C.M., Ahn S.H., Park D.J., Kim H.H. (2017). Laparoscopic gastrojejunostomy versus duodenal stenting in unresectable gastric cancer with gastric outlet obstruction. Ann. Surg. Treat. Res..

[22] Kobayashi S., Ueno M., Nagashima S., Sano Y., Kawano K., Fukushima T., Asama H., Tezuka S., Morimoto M. (2021). Association between time to stent dysfunction and the anti-tumour effect of systemic chemotherapy following stent placement in patients with pancreaticobiliary cancers and malignant gastric outlet obstruction: A retrospective cohort study. BMC Cancer.

[23] Yoshida Y., Fukutomi A., Tanaka M., Sugiura T., Kawata N., Kawai S., Kito Y., Hamauchi S., Tsushima T., Yokota T. (2017). Gastrojejunostomy versus duodenal stent placement for gastric outlet obstruction in patients with unresectable pancreatic cancer. Pancreatology.

[24] Matsumoto K., Kato H., Horiguchi S., Tsutsumi K., Saragai Y., Takada S., Mizukawa S., Muro S., Uchida D., Tomoda T. (2018). Efficacy and safety of chemotherapy after endoscopic double stenting for malignant duodenal and biliary obstructions in patients with advanced pancreatic cancer: A single-institution retrospective analysis. BMC Gastroenterol..

[25] Iqbal U., Khara H.S., Hu Y., Kumar V., Tufail K., Confer B., Diehl D.L. (2020). EUS-guided gastroenterostomy for the management of gastric outlet obstruction: A systematic review and meta-analysis. Endosc. Ultrasound.

[26] Chen Y.I., Itoi T., Baron T.H., Nieto J., Haito-Chavez Y., Grimm I.S., Ismail A., Ngamruenphong S., Bukhari M., Hajiyeva G. (2017). EUS-guided gastroenterostomy is comparable to enteral stenting with fewer re-interventions in malignant gastric outlet obstruction. Surg. Endosc..

[27] Chen Y.I., Kunda R., Storm A.C., Aridi H.D., Thompson C.C., Nieto J., James T., Irani S., Bukhari M., Gutierrez O.B. (2018). EUS-guided gastroenterostomy: A multicenter study comparing the direct and balloon-assisted techniques. Gastrointest. Endosc..

[28] Ge P.S., Young J.Y., Dong W., Thompson C.C. (2019). EUS-guided gastroenterostomy versus enteral stent placement for palliation of malignant gastric outlet obstruction. Surg. Endosc..

[29] Oikarinen H., Leinonen S., Karttunen A., Tikkakoski T., Hetemaa T., Mäkelä J., Päivänsalo M. (1999). Patency and complications of percutaneously inserted metallic stents in malignant biliary obstruction. J. Vasc. Interv. Radiol..

[30] Lee T.H., Choi J.H., Park D.H., Song T.J., Kim D.U., Paik W.H., Hwangbo Y., Lee S.S., Seo D.W., Lee S.K. (2016). Similar Efficacies of Endoscopic Ultrasound-guided Transmural and Percutaneous Drainage for Malignant Distal Biliary Obstruction. Clin. Gastroenterol. Hepatol..

[31] Wang K., Zhu J., Xing L., Wang Y., Jin Z., Li Z. (2016). Assessment of efficacy and safety of EUS-guided biliary drainage: A systematic review. Gastrointest. Endosc..

[32] Khashab M.A., Van der Merwe S., Kunda R., El Zein M.H., Teoh A.Y., Marson F.P., Fabbri C., Tarantino I., Varadarajulu S., Modayil R.J. (2016). Prospective international multicenter study on endoscopic ultrasound-guided biliary drainage for patients with malignant distal biliary obstruction after failed endoscopic retrograde cholangiopancreatography. Endosc. Int. Open.

[33] Park J.K., Woo Y.S., Noh D.H., Yang J.I., Bae S.Y., Yun H.S., Lee J.K., Lee K.T., Lee K.H. (2018). Efficacy of EUS-guided and ERCP-guided biliary drainage for malignant biliary obstruction: Prospective randomized controlled study. Gastrointest. Endosc..

[34] Bang J.Y., Navaneethan U., Hasan M., Hawes R., Varadarajulu S. (2018). Stent placement by EUS or ERCP for primary biliary decompression in pancreatic cancer: A randomized trial (with videos). Gastrointest. Endosc..

[35] Yamao K., Kitano M., Takenaka M., Minaga K., Sakurai T., Watanabe T., Kayahara T., Yoshikawa T., Yamashita Y., Asada M. (2018). Outcomes of endoscopic biliary drainage in pancreatic cancer patients with an indwelling gastroduodenal stent: A multicenter cohort study in West Japan. Gastrointest. Endosc..

[36] Hamada T., Isayama H., Nakai Y., Togawa O., Kogure H., Kawakubo K., Tsujino T., Sasahira N., Hirano K., Yamamoto N. (2014). Transmural biliary drainage can be an alternative to transpapillary drainage in patients with an indwelling duodenal stent. Dig. Dis. Sci..

[37] Hamada T., Isayama H., Nakai Y., Togawa O., Kogure H., Kawakubo K., Takeshi T., Sasahira N., Hirano K., Yamamoto N. (2011). Duodenal invasion is a risk factor for the early dysfunction of biliary metal stents in unresectable pancreatic cancer. Gastrointest. Endosc..

[38] Ogura T., Chiba Y., Masuda D., Kitano M., Sano T., Saori O., Yamamoto K., Imaoka H., Imoto A., Takeuchi T. (2016). Comparison of the clinical impact of endoscopic ultrasound-guided choledochoduodenostomy and hepaticogastrostomy for bile duct obstruction with duodenal obstruction. Endoscopy.

[39] Minaga K., Ogura T., Shiomi H., Imai H., Hoki N., Takenaka M., Nishikiori H., Yamashita Y., Hisa T., Kato H. (2019). Comparison of the efficacy and safety of endoscopic ultrasound-guided choledochoduodenostomy and hepaticogastrostomy for malignant distal biliary obstruction: Multicenter, randomized, clinical trial. Dig. Endosc..

[40] Kaw M., Singh S., Gagneja H. (2003). Clinical outcome of simultaneous self-expandable metal stents for palliation of malignant biliary and duodenal obstruction. Surg. Endosc..

[41] Vanbiervliet G., Demarquay J.F., Dumas R., Caroli-Bosc F.X., Piche T., Tran A. (2004). Endoscopic insertion of biliary stents in 18 patients with metallic duodenal stents who developed secondary malignant obstructive jaundice. Gastroenterol. Clin. Biol..

[42] Maire F., Hammel P., Ponsot P., Aubert A., O’toole D., Hentic O., Levy P., Ruszniewski P. (2006). Long-term outcome of biliary and duodenal stents in palliative treatment of patients with unresectable adenocarcinoma of the head of pancreas. Am. J. Gastroenterol..

[43] Khashab M.A., Valeshabad A.K., Leung W., Camilo J., Fukami N., Shieh F., Diehl D., Attam R., Vleggaar F.P., Saxena P. (2014). Multicenter experience with performance of ERCP in patients with an indwelling duodenal stent. Endoscopy.

[44] Yu J., Hao J., Wu D., Lang H. (2014). Retrospective evaluation of endoscopic stenting of combined malignant common bile duct and gastric outlet-duodenum obstructions. Exp. Ther. Med..

[45] Canena J., Coimbra J., Carvalho D., Rodrigues C., Silva M., Costa M., Horta D., Dias A.M., Seves I., Ramos G. (2014). Endoscopic bilio-duodenal bypass: Outcomes of primary and revision efficacy of combined metallic stents in malignant duodenal and biliary obstructions. Dig. Dis. Sci..

[46] Manta R., Conigliaro R., Mangiafico S., Forti E., Bertani H., Frazzoni M., Galloro G., Mutignani M., Zullo A. (2016). A multimodal, one-session endoscopic approach for management of patients with advanced pancreatic cancer. Surg. Endosc..

[47] Hori Y., Naitoh I., Hayashi K., Kondo H., Yoshida M., Shimizu S., Hirano A., Okumura F., Ando T., Jinno N. (2018). Covered duodenal self-expandable metal stents prolong biliary stent patency in double stenting: The largest series of bilioduodenal obstruction. J. Gastroenterol. Hepatol..

[48] Debourdeau A., Caillol F., Zemmour C., Winkler J.P., Decoster C., Pesenti C., Ratone J.P., Boher J.M., Giovannini M. (2021). Endoscopic management of concomitant biliary and duodenal malignant obstruction: Impact of the timing of drainage for one vs. two procedures and the modalities of biliary drainage. Endosc. Ultrasound.

[49] Mangiavillano B., Kunda R., Robles-Medranda C., Oleas R., Anderloni A., Sportes A., Fabbri C., Binda C., Auriemma F., Eusebi L.H. (2021). Lumen-apposing metal stent through the meshes of duodenal metal stents for palliation of malignant jaundice. Endosc. Int. Open.

[50] Boumitri C., Gaidhane M., Kahaleh M. (2016). Antireflux metallic biliary stents: Where do we stand?. Gastrointest. Endosc..

[51] Dua K.S., Reddy N.D., Rao V.G., Banerjee R., Medda B., Lang I. (2007). Impact of reducing duodenobiliary reflux on biliary stent patency: An in vitro evaluation and a prospective randomized clinical trial that used a biliary stent with an antireflux valve. Gastrointest. Endosc..

[52] Lee K.J., Chung M.J., Park J.Y., Lee D.H., Jung S., Bang B.W., Park S.W., Chung J.B., Song S.Y., Bang S. (2013). Clinical advantages of a metal stent with an S-shaped anti-reflux valve in malignant biliary obstruction. Dig. Endosc..

[53] Hu B., Wang T.T., Wu J., Shi Z.M., Gao D.J., Pan Y.M. (2014). Antireflux stents to reduce the risk of cholangitis in patients with malignant biliary strictures: A randomized trial. Endoscopy.

[54] Lee Y.N., Moon J.H., Choi H.J., Choi M.H., Lee T.H., Cha S.W., Cho Y.D., Choi S.Y., Lee H.K., Park S.H. (2016). Effectiveness of a newly designed antireflux valve metal stent to reduce duodenobiliary reflux in patients with unresectable distal malignant biliary obstruction: A randomized, controlled pilot study (with videos). Gastrointest. Endosc..

[55] Hamada T., Isayama H., Nakai Y., Iwashita T., Ito Y., Mukai T., Yagioka H., Saito T., Togawa O., Ryozawa S. (2019). Antireflux covered metal stent for nonresectable distal malignant biliary obstruction: Multicenter randomized controlled trial. Dig. Endosc..

[56] Hamada T., Nakai Y., Isayama H., Koike K. (2021). Antireflux metal stent for biliary obstruction: Any benefits?. Dig. Endosc..

[57] Kin T., Ishii K., Okabe Y., Itoi T., Katanuma A. (2021). Feasibility of biliary stenting to distal malignant biliary obstruction using a novel designed metal stent with duckbill-shaped anti-reflux valve. Dig. Endosc..

[58] Yamada Y., Sasaki T., Takeda T., Mie T., Furukawa T., Kasuga A., Matsuyama M., Ozaka M., Igarashi Y., Sasahira N. (2021). A novel laser-cut fully covered metal stent with anti-reflux valve in patients with malignant distal biliary obstruction refractory to conventional covered metal stent. J. Hepatobiliary Pancreat Sci..

[59] Sasaki T., Takeda T., Sasahira N. (2020). Double stenting with EUS-CDS using a new anti-reflux metal stent for combined malignant biliary and duodenal obstruction. J. Hepatobiliary Pancreat Sci..

[60] Binmoeller K.F., Shah J. (2011). A novel lumen-apposing stent for transluminal drainage of nonadherent extraintestinal fluid collections. Endoscopy.

[61] Mussetto A., Fugazza A., Fuccio L., Triossi O., Repici A., Anderloni A. (2018). Current uses and outcomes of lumen-apposing metal stents. Ann. Gastroenterol..

[62] Khashab M.A., Kumbhari V., Grimm I.S., Ngamruengphong S., Aguila G., El Zein M., Kalloo A.N., Baron T.H. (2015). EUS-guided gastroenterostomy: The first U.S. clinical experience (with video). Gastrointest. Endosc..

[63] Itoi T., Ishii K., Ikeuchi N., Sofuni A., Gotoda T., Moriyasu F., Dhir V., Teoh A.Y.B., Binmoeller K.F. (2016). Prospective evaluation of endoscopic ultrasonography-guided double-balloon-occluded gastrojejunostomy bypass (EPASS) for malignant gastric outlet obstruction. Gut.

[64] Irani S., Itoi T., Baron T.H., Khashab M. (2020). EUS-guided gastroenterostomy: Techniques from East to West. VideoGIE.

[65] Itoi T., Baron T.H., Khashab M.A., Tsuchiya T., Irani S., Dhir V., Bun Teoh A.Y. (2017). Technical review of endoscopic ultrasonography-guided gastroenterostomy in 2017. Dig. Endosc..

[66] Marino A., Bessissow A., Miller C., Valenti D., Boucher L., Chaudhury P., Barkun J., Forbes N., Khashab M.A., Martel M. (2021). Modified endoscopic ultrasound-guided double-balloon-occluded gastroenterostomy bypass (M-EPASS): A pilot study. Endoscopy.

[67] Bronswijk M., Vanella G., van Malenstein H., Laleman W., Jaekers J., Topal B., Daams F., Besselink M.G., Arcidiacono P.G., Voermans R.P. (2021). Laparoscopic versus EUS-guided gastroenterostomy for gastric outlet obstruction: An international multicenter propensity score-matched comparison (with video). Gastrointest. Endosc..

[68] Chandan S., Khan S.R., Mohan B.P., Shah A.R., Bilal M., Ramai D., Bhogal N., Dhindsa B., Kassab L.L., Singh S. (2021). EUS-guided gastroenterostomy versus enteral stenting for gastric outlet obstruction: Systematic review and meta-analysis. Endosc. Int. Open.

[69] Kongkam P., Luangsukrerk T., Harinwan K., Vanduangden K., Plaidum S., Rerknimitr R., Kullavanijaya P. (2020). Combination of endoscopic-ultrasound guided choledochoduodenostomy and gastrojejunostomy resolving combined distal biliary and duodenal obstruction. Endoscopy.

[70] Lajin M. (2020). EUS-guided choledocoduodenostomy and gastroenterostomy to palliate simultaneous biliary and duodenal obstruction due to pancreatic cancer. Endosc. Int. Open.

[71] Platt K.D., Bhalla S., Sondhi A.R., Millet J.D., Law R.J. (2021). EUS-guided gastrojejunostomy and hepaticogastrostomy for malignant duodenal and biliary obstruction. VideoGIE.

[72] Imai H., Takenaka M., Omoto S., Kamata K., Miyata T., Minaga K., Yamao K., Sakurai T., Nishida N., Watanabe T. (2017). Utility of Endoscopic Ultrasound-Guided Hepaticogastrostomy with Antegrade Stenting for Malignant Biliary Obstruction after Failed Endoscopic Retrograde Cholangiopancreatography. Oncology.

[73] Yamamoto K., Itoi T., Tsuchiya T., Tanaka R., Tonozuka R., Honjo M., Mukai S., Fujita M., Asai Y., Matsunami Y. (2018). EUS-guided antegrade metal stenting with hepaticoenterostomy using a dedicated plastic stent with a review of the literature (with video). Endosc. Ultrasound.

